# Symptoms and other factors associated with time to diagnosis and stage of lung cancer: a prospective cohort study

**DOI:** 10.1038/bjc.2015.30

**Published:** 2015-03-03

**Authors:** F M Walter, G Rubin, C Bankhead, H C Morris, N Hall, K Mills, C Dobson, R C Rintoul, W Hamilton, J Emery

**Affiliations:** 1 Department of Public Health & Primary Care, University of Cambridge, Cambridge CB1 8RN, UK; 2 General Practice & Primary Care Academic Centre, University of Melbourne, Melbourne, Victoria, Australia; 3 School of Medicine, Pharmacy & Health, Durham University, Wolfson Building, Stockton on Tees TS17 6BH, UK; 4 Nuffield Department of Primary Care Health Sciences, University of Oxford, Oxford OX2 6GG, UK; 5 Department of Thoracic Oncology, Papworth Hospital NHS Foundation Trust, Cambridge CB23 3RE, UK; 6 University of Exeter, College House, St Luke's Campus, Exeter EX2 4TE, UK; 7 Department of General Practice, University of Western Australia, Perth, Western Australia, Australia

**Keywords:** lung cancer, symptom, diagnosis, diagnostic interval, early stage, late stage

## Abstract

**Background:**

This prospective cohort study aimed to identify symptom and patient factors that influence time to lung cancer diagnosis and stage at diagnosis.

**Methods:**

Data relating to symptoms were collected from patients upon referral with symptoms suspicious of lung cancer in two English regions; we also examined primary care and hospital records for diagnostic routes and diagnoses. Descriptive and regression analyses were used to investigate associations between symptoms and patient factors with diagnostic intervals and stage.

**Results:**

Among 963 participants, 15.9% were diagnosed with primary lung cancer, 5.9% with other thoracic malignancies and 78.2% with non-malignant conditions. Only half the cohort had an isolated first symptom (475, 49.3%); synchronous first symptoms were common. Haemoptysis, reported by 21.6% of cases, was the only initial symptom associated with cancer. Diagnostic intervals were shorter for cancer than non-cancer diagnoses (91 *vs* 124 days, *P*=0.037) and for late-stage than early-stage cancer (106 *vs* 168 days, *P*=0.02). Chest/shoulder pain was the only first symptom with a shorter diagnostic interval for cancer compared with non-cancer diagnoses (*P*=0.003).

**Conclusions:**

Haemoptysis is the strongest symptom predictor of lung cancer but occurs in only a fifth of patients. Programmes for expediting earlier diagnosis need to focus on multiple symptoms and their evolution.

Lung cancer is the most common cancer worldwide. Most cases are diagnosed in symptomatic patients; the majority have late-stage disease and a poor prognosis (Cancer Research UK, 2014a). In the United Kingdom, lung cancer is the most common cause of cancer mortality (Cancer Research UK, 2014b). Fewer than 10% of those diagnosed with lung cancer survive for 5 years, and UK lung cancer patients have poorer survival than those in other countries (Abdel-Rahman *et al*, 2009). This may partly be due to longer time between the onset of cancer symptoms and the patient's presentation to health care, leading to more late-stage diagnoses and therefore less eligibility for potentially curative treatment (Holmberg *et al*, 2010). Late-stage disease at diagnosis is associated with socioeconomic deprivation, especially among older men, and those with 20 or more pack-years of smoking, even if they stopped smoking within the previous 10 years (Lyratzopoulos *et al*, 2012). In England there are also regional differences that may reflect socioeconomic deprivation in northern compared with southern regions.

Lung cancer patients are often symptomatic for many months before presentation, irrespective of their disease stage at diagnosis (Corner *et al*, 2005). They commonly experience multiple symptoms, both lung-specific (cough, breathing changes, chest pain and haemoptysis) and systemic (loss of weight or appetite, fatigue) (Corner *et al*, 2005; Hamilton *et al*, 2005). Those most at risk may not interpret their initial symptoms as serious, or may attribute them to ageing, lifestyle, smoking habits or other comorbidities (Corner *et al*, 2005, 2006; Brindle *et al*, 2012). International comparisons suggest that UK differences in cancer awareness and beliefs may contribute to later presentation (Forbes *et al*, 2013), and there is early evidence that approaches to improve symptom awareness result in earlier-stage lung cancer diagnosis, as well as increased numbers of chest X-rays and total lung cancer diagnoses (Athey *et al*, 2012).

In primary care, general practitioners (GPs) face similar difficulties in evaluating new or evolving symptoms suspicious of lung cancer. One-third of lung cancer patients have three or more pre-referral consultations compared with only 3% of patients diagnosed with breast cancer (Lyratzopoulos *et al*, 2013). Furthermore, the pathway to diagnosis in primary care may be complex, and delays may occur with presentation complicated by comorbidity, false negative chest X-ray reports and delayed or declined referral (Mitchell *et al*, 2013; Rubin *et al*, 2014).

Much of the evidence about which symptoms best predict cancer or are associated with later diagnosis comes from retrospective studies in people with a lung cancer diagnosis or from general practice data sets, which are limited by issues of data recording. Little is known about the diagnostic pathways of those with similar symptoms but ultimately other malignant and non-malignant diagnoses. Less is known about which symptoms result in prompt or less timely diagnosis. We therefore recruited a prospective cohort of patients in two English regions at the point of their referral for suspected lung cancer. We aimed to investigate the symptoms and other clinical and sociodemographic factors associated with lung cancer diagnosis, time to diagnosis and stage at diagnosis.

## Methods

### Setting and governance

We recruited patients in the East and North East of England who were referred to four secondary care (East 1, North East 3) and one tertiary care (East) hospital between December 2010 and December 2012. We gained appropriate ethics (reference: 10/H0306/50) and clinical governance approvals. The SYMPTOM lung study was conducted alongside the SYMPTOM colorectal and pancreas studies, collectively part of the NIHR-funded DISCOVERY programme of applied research.

### Patient recruitment

All referral letters to urgent and routine respiratory clinics across the five sites were reviewed by a research nurse. Patients aged ⩾40 years with any symptoms suspicious of lung cancer were sent a study pack; this included an information sheet, the SYMPTOM lung questionnaire and a freepost envelope to return the completed questionnaire to the research team. Exclusion criteria included people already undergoing treatment for any cancer (excluding non-melanotic skin cancer) and those with serious mental and/or physical disease. Patients were only approached on a single occasion; no follow-up letters were sent.

### Data collection

Our approaches to data collection, analysis and reporting were based on the recommendations of the Aarhus statement for the conduct of cancer diagnostic studies (Weller *et al*, 2012).

#### Patient data

The SYMPTOM lung questionnaire, drawing on the C-SIM questionnaire (Neal *et al*, 2014b), was modified for use among people before diagnosis. Due to the sensitive nature of the subject matter, we consulted widely among clinical and research colleagues and patient representatives to achieve appropriate wording. The questionnaire starts with the question ‘What was the first thing or symptom you noticed that made you think something might be wrong?' followed by nine specific symptoms (coughing up blood; cough or worsening of a long-standing cough >3 weeks, breathlessness >3 weeks, chest/shoulder pain >3 weeks, hoarseness >3 weeks; plus decreased appetite, unexplained weight loss, fatigue/tiredness, feeling different ‘in yourself'). Exact or estimated dates were requested for all symptoms. The remaining sections contained items about other symptoms, and demographic and clinical details.

#### Primary care data

GPs completed a proforma from their clinical records, providing dates of the first presentation with any symptom listed in the SYMPTOM questionnaire within the previous 2 years, plus its duration before presentation, if recorded.

#### Hospital data

Study researchers extracted data from hospital medical records, including date of referral and route (urgent, routine, emergency, other); date of first consultation; investigations and findings; and diagnosis and date (histological, clinical, MDT meetings). Proformas were completed up to 6 months after recruitment to allow sufficient time for completion of investigation and initiation of treatment. Across both geographical sites, double data abstraction of a 5% sample of hospital data (dates of referral, first appointment, diagnosis and stage) confirmed an acceptable level of agreement (>80% for dates; >90% for diagnosis and stage).

### Data handling

#### Clinical outcomes

The date of diagnosis was based on the date on the pathology report where possible; the first date of clinical diagnosis in the medical record was used where pathology was unavailable. Participants were classified into three groups: those with primary lung cancer (LC), other cancer (OC) and no cancer (NC). The main analyses focused on LC *vs* NC, with secondary analyses including all cancers (LC plus OC) *vs* NC. Primary lung cancer staging was categorised using TNM status at diagnosis (Travis *et al*, 2011), and further categorised into early-stage (stages I and II) and late-stage (stages III and IV). Difficult or unusual diagnoses, or cases with incomplete data, were agreed by an expert clinical consensus group (FMW, JE, GR, RCR).

#### Demographic and clinical variables

Demographic details collected in the patient questionnaire included: gender; age (treated as a continuous variable); ethnicity (coded as white *vs* non-white); smoking status; educational status; occupational status; living alone; and postcode, used to derive national quintiles according to the Index of Multiple Deprivation (IMD) (1 ‘least deprived' to 5 ‘most deprived'). Clinical variables relating to comorbidities included respiratory disease (chronic obstructive pulmonary disease (COPD)/asthma/other lung disease), anxiety/depression, heart disease, diabetes and arthritis. A family history of cancer was also recorded (present *vs* absent).

#### Symptoms

Symptoms >2 years before diagnosis date were omitted from analysis, as we considered these unlikely to be associated with the developing disease (Ades *et al*, 2014); all other reported symptoms were included in analysis. Participants' estimated dates were converted by adapting an algorithm used in the C-SIM trial (Neal *et al*, 2014b). In brief, the mid-month date was used for ‘a month' mid-year for ‘a year' mid-April, mid-July, mid-October and mid-January for the seasons; and the actual dates for Christmas and Easter. We devised a second set of rules to allow for the combination of exact and estimated dates. If responses to the unprompted first symptom question matched a specific question, they were given the corresponding codes. The initial symptom was then identified for each participant. Many participants reported more than one initial symptom, termed ‘synchronous first symptoms'. The initial and synchronous first symptoms were combined as ‘first symptom/s'.

#### The total diagnostic interval

The total diagnostic interval (TDI), or ‘time to diagnosis', defined as the time from the first symptom/s to the date of diagnosis, was calculated for all participants.

### Analysis

Descriptive analyses were performed on all demographic, clinical and symptom data for the group as a whole, and by the diagnostic group (LC, OC and NC). The LC group was also described according to the cancer stage at diagnosis. Clinically relevant *a priori* demographics, comorbidities, first symptom/s and family history of cancer variables, and those significant at the 20% level in univariate analysis, were included in multivariate analyses. The referral variable, and those with fewer than 10 cases, were excluded. Logistic regression or Cox regression analyses were performed as appropriate. Sensitivity analyses were performed alongside each primary analysis to examine (i) all cancers (LC plus OC) *vs* NC, and (ii) the ‘waiting time paradox' by excluding cases in which diagnosis occurred within 28 days of first symptom/s (Tørring *et al*, 2012).

## Results

A total of 5097 patients were approached and 995 were recruited, giving an overall 19.5% response rate (East 25.1%, North East 16.8%). The demographics of the responders were similar to those of non-responders (responders 54% male, median age 67 years; non-responders 49.7% male, median age 66 years). The disease stage distribution of our cohort was comparable to national data (late stage 68.2 *vs* 67.6%) (Cancer Research UK, 2014b). Twenty participants were excluded (returning questionnaire >3 months after diagnosis *n*=8, not meeting recruitment criteria *n*=4, recent metastatic disease *n*=2, recruited via screening trial *n*=1, no consent given to access hospital records *n*=5), and there were insufficient data for analysis for a further 12, leaving a final cohort of 963 participants.

### Descriptive

The characteristics of the whole cohort are provided in Table 1. 153 (15.9%) participants were diagnosed with primary lung cancer, 57 (5.9%) with other thoracic malignancies (metastases from extra-thoracic primaries *n*=21; malignant mesothelioma *n*=19, carcinoid *n*=7, lymphoma *n*=7 and other *n*=3), and 753 (78.2%) with no cancer (nil abnormal detected *n*=251, COPD *n*=51, asthma *n*=37, sarcoidosis *n*=14, other (infection and so on) *n*=396 and missing *n*=4). The majority of those with primary lung cancer had late-stage disease (*n*=103, 68.2% early-stage disease *n*=48, 31.8%). Only 2 (1.3%) lung cancers were unstaged. Compared with people diagnosed with no cancer, those diagnosed with primary lung cancer were more likely to be older (*P*<0.001), retired (*P*=0.013) and to have attained a lower educational level (*P*=0.002). They were also more likely to be current or ex-smokers (*P*<0.001) and to live alone (*P*=0.019). We found no significant differences in deprivation levels or ethnicity between the diagnostic groups.

Among the total cohort, only half had an isolated first symptom (475, 49.3%). Synchronous first symptoms were common, with 19.0% having two first symptoms, 8.8% having three and >10% with four or more synchronous first symptoms. 12.5% reported no symptoms within 2 years of diagnosis. Cough or worsening of a long-standing cough and breathlessness or worsening of long-standing breathlessness were the most common symptoms, and for each symptom more were reported as ‘all symptoms' than ‘first symptoms' suggesting the evolution of symptoms over time (Table 2). Symptoms not specifically mentioned in the questionnaire, such as backache, sickness/indigestion and symptoms of acute respiratory illness, were individually reported by fewer than 10% of participants. Coughing up blood and unexplained weight loss at any time were infrequently reported in the whole cohort (13.5 and 11.1%, respectively) but were the only symptoms reported by significantly more people diagnosed with lung cancer than with no cancer (21.6 *vs* 11.8%, *P*=0.001; 15 *vs* 9.2%, *P*=0.028, respectively). Cough or worsening of a long-standing cough was reported less commonly as a first symptom in people with lung cancer (39.9 *vs* 50.7%, *P*=0.014).The median TDI across the whole cohort was 117 days (IQR 50–269); people diagnosed with lung cancer had a significantly shorter median TDI than people diagnosed with no cancer (LC 91 *vs* NC 124 days, *P*=0.037) (Table 3). Chest/shoulder pain was the only first symptom associated with a shorter median TDI for lung cancer (LC 69 *vs* NC 120 days, *P*=0.044). For early-stage lung cancer, the median TDI for any symptom was 141 days compared with 87 days for late-stage lung cancer (*P*=0.33). After adjustment for the ‘waiting time paradox' those with late-stage lung cancer had a significantly shorter TDI (early stage 168 *vs* late stage 106 days, *P*=0.02) (Supplementary Online Material A1).

### Regression analyses

Coughing up blood, cough or worsening cough and decreased appetite were significant predictors of shorter TDI for the total cohort (all *P*<0.001), whereas a respiratory comorbidity was associated with longer TDI (*P*=0.04) (Table 4). Chest/shoulder pain was associated with shorter TDI for primary lung cancer (*P*=0.03). When the regression analyses were repeated for any cancer, lower educational level was also associated with longer TDI (*P*=0.003). For early-stage lung cancer, a self-reported family history of cancer and breathlessness or worsening breathlessness were associated with shorter TDI (Table 5). Increasing age and coughing up blood were the only factors that predicted lung cancer (Table 6 and Supplementary Online Material Table A2). Never smoking, or ex-smoking, were inversely associated with the risk of a lung cancer diagnosis, and demonstrated a dose–response relationship. Both arthritis and other respiratory diseases were associated with a lower risk of lung cancer. These factors remained significant in the regression analyses; however, the model explained only 15% of total variability.

## Discussion

This is one of the first studies worldwide to study diagnostic pathways for lung cancer by recruiting before diagnosis a large prospective cohort of patients with suspicious symptoms. Haemoptysis was the only symptom associated with lung cancer, but it occurred in just 21.6% of cases, and only 4.6% of cases as a first symptom; other associated factors were increasing age, smoking status and respiratory and arthritis comorbidities. Diagnostic intervals were longer for non-cancer than cancer diagnoses and for early-stage than late-stage lung cancer. Prolonged chest/shoulder pain was the only first symptom associated with a shorter diagnostic interval for lung cancer than for non-cancer diagnoses. Our findings show that people referred with symptoms suspicious of lung cancer often have complex symptomatology. Only half of our cohort reported an isolated first symptom; the majority developed multiple symptoms over time. This study set out to investigate the TDI from perception of first symptom to diagnosis; therefore, our unit of analysis was the initial symptom or first synchronous symptoms. Although this approach allows us to make robust comparisons with evidence reported from England and elsewhere, it may obscure the finer detail of symptom patterns and clusters as they evolve over time, and their effects on timely help-seeking by patients and timely diagnosis in primary and secondary care.

We found that people with lung cancer were diagnosed more quickly than those with an alternative diagnosis. This may reflect the guidance on urgent referral for suspected lung cancer in England (National Institute for Health and Care Excellence, 2005), which recommends that ‘alarm' symptoms such as haemoptysis warrant urgent chest X-ray and referral. Symptoms other than haemoptysis in this relatively large prospective cohort study did not help differentiate lung cancer from other diagnoses, even though some, such as weight loss, can be indicative of advanced disease. This highlights the challenge for earlier detection in primary care for patients with less specific symptoms (Shim *et al*, 2014).

The median TDI for any symptom was 117 days, and 91 days for those with lung cancer. This remains a substantial period between a person first noticing a symptom and receiving a diagnosis. It is worth noting that a national ‘Be Clear on Cancer' lung cancer campaign ran for 2 months (May and June 2012) during our recruitment period (Cancer Research UK, 2014c). The diagnostic intervals are broadly similar to evidence from a UK General Practice Research Database analysis (Neal *et al*, 2014a). Secondary analyses of a national audit of cancer diagnosis from primary care medical records also suggest that the symptoms and signs of lung cancer may be more quickly acted upon by patients than GPs: lung cancer patients had a median patient interval of just 12 days (Keeble *et al*, 2014), whereas more than 30% of lung cancer patients had three or more primary care consultations before referral (Lyratzopoulos *et al*, 2013).

In common with a Danish prospective population-based study of diagnostic intervals (Tørring *et al*, 2013), we found shorter median intervals associated with later stage at diagnosis, even after adjusting for the ‘waiting time paradox'. This adjustment aims to account for patients who present with very short intervals and severe symptoms associated with late-stage disease, often presenting to emergency departments (Tørring *et al*, 2012). However, the problem of confounding remains to some extent even after this adjustment; late-stage disease may have different symptom profiles that affect help-seeking and diagnostic pathways. This suggests that tumour factors (such as histological type and location) and host factors (such as comorbidity) could influence diagnostic intervals and result in apparently earlier diagnosis of later-stage disease (Tørring *et al*, 2013).

Having respiratory comorbidity increased time to diagnosis across the cohort, but had a lower risk of being diagnosed with lung cancer, suggesting that the respiratory symptoms were associated with that comorbidity rather than lung cancer. The longer diagnostic intervals in people with respiratory comorbidities may be owing to the patient and their GP attributing new or worsening symptoms to pre-existing illness (Emery *et al*, 2013; Birt *et al*, 2014). Persistent or worsening chest/shoulder pain was associated with a shorter time to cancer diagnosis, possibly because the symptom triggered an urgent referral or admission to hospital to exclude cardiovascular causes.

Key strengths of this study are the prospective design and the collection of data from several sources: patient reports, and primary care and specialist records. The analytical and reporting approaches were robust and performed according to the methodological approaches and definitions recommended in the Aarhus statement (Weller *et al*, 2012). We chose to define the date of first symptom/s using the patient-reported date rather than the primary care-reported date, as we were analysing the patient-reported symptom/s. Ideally, a study would recruit patients from primary care before referral; however, this would be accompanied by major logistical and resource implications of identifying a prospective cohort in primary care with respiratory symptoms with sufficient numbers of cancers. Instead, we recruited patients when first encountered in secondary care; this had the added benefit of recruiting patients admitted via the emergency route. Recruitment involved two regions of England, selected to ensure a broad range of socioeconomic, educational and occupational levels. The deprivation data suggest that the cohort was representative of the national population.

The main study limitation is the recruitment rate of 19.5% overall, ranging from 17% in the North East to 25% in the East of England, similar to other recent studies (McRonald *et al*, 2014). It is possible that many of the target populations were unable or unwilling to complete a questionnaire because they were coping with a serious diagnosis or undergoing treatment, regardless of final diagnosis. However, the demographic of our non-responders were very similar to those in the cohort, and the proportion of late-stage lung cancer was identical to national data. This suggests that we did not recruit a healthier cohort, and our findings are likely to be generalisable. Although this was a large cohort, we had insufficient power to examine specific clusters of symptoms and their associations with our outcomes; a much larger prospective study would be required to achieve this. The analyses focused only on the first symptom or symptoms. The impact of subsequent symptoms on time to diagnosis requires further study. This paper reports our data on the TDI and factors associated with this. Future analyses will explore the relative contributions of the patient interval (from first symptom/s to first presentation in primary care), the primary care interval (from first presentation to referral) and the secondary care interval (from referral to diagnosis) to the TDI.

In conclusion, identifying symptoms and other factors that should prompt an individual to seek help or a GP to perform the appropriate diagnostic test or refer appropriately remains challenging. Haemoptysis is the most important symptom associated with lung cancer, but this is reported as the first symptom in less than 5% of cases. Despite conducting such a large prospective cohort study, we failed to identify any other strong signals of lung cancer diagnosis. This is an important finding while we await the revised National Institute for Health and Care Excellence guidelines for early detection of lung cancer. This study suggests that lung cancer awareness campaigns that currently concentrate on a single symptom should instead consider messages that reflect the multi-symptom nature of its presentation. It may also be that targeted interventions at high-risk populations aimed at symptom monitoring could be more effective at recognising symptom evolution (Smith *et al*, 2013). Policy initiatives such as prompt chest X-rays for high-risk groups, and the increasingly widespread use of clinical decision support (Hamilton *et al*, 2013), can be informed by our findings. The next step is to understand the potentially subtle differences in impact of symptoms and patient factors on the patient, GP and specialist intervals. These data will provide support for more targeted evaluation of suspicious symptoms in an attempt to identify lung cancer at an earlier and more amenable stage.

## Figures and Tables

**Table 1:** Characteristics of participants

**Characteristic**	**Primary lung cancer (LC) (*****n*****=153)**^a^	**No cancer** **(NC) (*****n*****=753)**	**Other lung cancer**^b^ **(OC) (*****n*****=57)**	**Total cohort (*****n*****=963)**
**Gender**				
Male	92 (60.1%)	393 (52.2%)	37 (64.9%)	522(54.2%)
Female	61 (39.9%)	360 (47.8%)	20 (35.1%)	441(45.8%)
Age*				
Median, range	70 (43–89)	65 (40–95)	71 (42–88)	66 (40–95)
Employment status*				
Employed	31 (20.3%)	230 (30.5%)	13 (22.8%)	274 (28.5%)
Unemployed	1 (0.7%)	17 (2.3%)	0 (0.0%)	18 (1.9%)
Retired	109 (71.2%)	424 (56.3%)	37 (64.9%)	570 (59.2%)
Sick/disabled	5 (3.2%)	31 (4.1%)	2 (3.5%)	38 (4.00%)
Other (including missing)	7 (4.6%)	51 (6.8%)	5 (8.8%)	63 (6.6%)
Highest education level*				
Degree/diploma/equivalent	38 (24.8%)	250 (33.2%)	20 (35.1%)	308 (32.0%)
A level/GCSE/O level	44 (28.8%)	264 (35.1%)	18 (31.6%)	326 (33.9%)
Other/none	71 (46.4%)	239 (31.7%)	19 (33.3%)	329 (34.2%)
**Ethnicity**				
White	151 (98.7%)	725 (96.3%)	55 (96.5%)	931 (96.7%)
Smoking status*^c^				
Current	36 (23.6%)	75 (10.0%)	4 (7.0%)	115 (11.9%)
Ex-smoker	105 (68.6%)	377 (50.1%)	35 (61.4%)	517 (53.7%)
Never	11 (7.2%)	283 (37.6%)	17 (29.8%)	311 (32.3%)
Lives alone*^d^				
Yes	48 (31.4%)	158 (21.0%)	12 (21.1%)	218 (22.6%)
No	104 (68.0%)	583 (77.4%)	44 (77.2%)	731 (75.9%)
**Deprivation (IMD—quintiles)**				
1st (least deprived)	40 (26.1%)	258 (34.3%)	19 (33.3%)	317 (32.9%)
2nd	40 (26.1%)	157 (20.9%)	19 (33.3%)	216 (22.4%)
3rd	26 (17.0%)	124 (16.5%)	10 (15.5%)	160 (16.6%)
4th	28 (18.3%)	96 (12.8%)	2 (3.5%)	126 (13.1%)
5th (most deprived)	19 (12.4%)	117 (15.5%)	7 (12.3%)	143 (14.9%)

Abbreviation: IMD=Index of Multiple Deprivation.

Values are *n* (%) unless otherwise stated. *P-value <0.05 for LC *vs* NC.

a Early-stage disease, *n*=48 (31.8%): stage IA, *n*=15 (9.8%); stage IB, *n*=14 (9.2%); stage IIA, *n*=11 (7.2%); stage IIB, *n*=8 (5.2%). Late-stage disease, *n*=103 (68.2%): stage IIIA, *n*=21 (13.7%); stage IIIB, *n*=19 (12.4%); stage IV, *n*=63 (41.2%).

b Metastases from extra-thoracic primaries, *n*=21; malignant mesothelioma, *n*=19; carcinoid, *n*=7; lymphoma, *n*=7; other, *n*=3.

c Smoking status—missing: primary lung cancer, *n*=1 (0.7%); no cancer, *n*=18 (2.4%); other lung cancer, *n*=1 (1.8%); total, *n*=20 (2.1%).

d Lives alone—missing: primary lung cancer, *n*=1 (0.7%); no cancer, *n*=12 (1.6%); other lung cancer, *n*=1 (1.8%); total *n*=14 (1.5%).

**Table 2:** Symptoms reported by participants, stratified by all and first symptom/s and diagnostic group

	**Primary lung cancer (LC) (*****n*****=153)**		**No cancer (NC) (*****n*****=753)**		**Other lung cancer** **(OC) (*****n*****=57**)		**Total cohort (*****n*****=963)**	
**Symptom**	**All symptoms**	**First symptoms**	**All symptoms**	**First symptoms**	**All symptoms**	**First symptoms**	**All symptoms**	**First symptoms**
**Symptoms in questionnaire**								
Coughing up blood	**33 (21.6%)**^a^	7 (4.6%)	89 (11.8%)	42 (5.6%)	8 (14.4%)	4 (7.0%)	130 (13.5%)	53 (5.5%)
Cough or worsening cough >3 weeks	86 (56.2%)	**61 (39.9%)**^b^	478 (63.5%)	382 (50.7%)	19 (33.3%)	16 (28.1%)	583 (60.5%)	459 (47.7%)
Breathlessness or worsening >3 weeks	63 (41.2%)	40 (26.1%)	324 (43.0%)	199 (26.4%)	27 (47.4%)	17 (29.8%)	414 (43.0%)	256 (26.6%)
Chest/shoulder pain >3 weeks	54 (35.3%)	24 (15.7%)	216 (28.7%)	110 (14.6%)	14 (24.6%)	8 (14.1%)	284 (29.5%)	142 (14.8%)
Hoarseness >3 weeks	19 (12.4%)	10 (6.5%)	137 (18.2%)	75 (10.0%)	5 (8.8%)	4 (7.0%)	161 (16.7%)	89 (9.2%)
Decreased appetite	34 (22.2%)	18 (11.8%)	123 (16.3%)	60 (8.0%)	20 (35.1%)	9 (15.8%)	177 (18.4%)	87 (9.0%)
Unexplained weight loss	**23 (15.0%)**^a^	11 (7.2%)	69 (9.2%)	30 (4.0%)	15 (26.3%)	5 (8.8%)	107 (11.1%)	46 (4.8%)
Fatigue or tiredness ‘unusual for you'	69 (45.1%)	40 (26.1%)	301 (40.0%)	179 (23.8%)	24 (42.1%)	12 (21.1%)	394 (40.9%)	231 (24.0%)
Different ‘in yourself'	53 (34.6%)	28 (18.3%)	279 (37.1%)	168 (22.3%)	28 (49.2%)	16 (28.1%)	360 (37.4%)	212 (22.1%)
**Other symptoms reported by participants**								
Backache	4 (2.6%)	7 (4.6%)	11 (1.5%)	46 (6.1%)	0 (0.0%)	0 (0.0%)	15 (1.6%)	53 (5.5%)
Chest infection, respiratory illness	0 (0.0%)	5 (3.3%)	19 (2.5%)	37 (4.9%)	0 (0.0%)	3 (5.3%)	19 (2.0%)	45 (4.7%)
Sickness, indigestion	0 (0.0%)	0 (0.0%)	6 (0.8%)	16 (2.1%)	0 (0.0%)	0 (0.0%)	6 (0.6%)	16 (1.7%)
Difficulty swallowing	0 (0.0%)	2 (1.3%)	2 (0.3%)	6 (0.8%)	0 (0.0%)	1 (1.8%)	2 (0.2%)	9 (0.9%)
Pain (excluding chest, shoulder and back)	2 (1.3%)	3 (2.0%)	6 (0.8%)	4 (0.5%)	1 (1.8%)	1 (1.8%)	9 (0.9%)	8 (0.8%)
Faintness, dizziness	0 (0.0%)	0 (0.0%)	3 (0.4%)	6 (0.8%)	0 (0.0%)	0 (0.0%)	3 (0.3%)	6 (0.6%)
Congestion, phlegm and sore throat	4 (2.6%)	**2 (1.3%)**^b^	58 (7.7%)	3 (0.4%)	0 (0.0%)	0 (0.0%)	62 (6.4%)	5 (0.5%)
Incidental radiology finding	3 (2.0%)	0 (0.0%)	4 (0.5%)	3 (0.4%)	1 (1.8%)	0 (0.0%)	8 (0.8%)	3 (0.3%)
Feverish, unwell	7 (4.6%)	0 (0.0%)	46 (6.1%)	2 (0.3%)	0 (0.0%)	0 (0.0%)	53 (5.5%)	2 (0.2%)
Other symptoms	3 (2.0%)	7 (4.6%)	13 (1.7%)	42 (5.6%)	3 (5.3%)	4 (7.0%)	19 (2.0%)	53 (5.5%)
Other illness (non-respiratory)	**2 (1.3%)**^a^	2 (1.3%)	0 (0.0%)	34 (4.5%)	0 (0.0%)	0 (0.0%)	2 (0.2%)	36 (3.7%)

Values are *n* (%) unless otherwise stated, and all columns add up to >100% because of multiple symptoms.

a *P*-value <0.05 for all symptoms for LC *vs* NC.

b *P*-value <0.05 for first symptom/s for LC *vs* NC.

**Table 3:** Total diagnostic interval for first symptom/s for total cohort and by diagnostic groups

	**Total cohort**^a^ (***n*****=963)**			**Primary lung cancer (LC) (*****n*****=153)**			**No cancer (NC) (*****n*****=753)**			
**Symptom**	**Median**	**IQR**	***n***	**Median**	**IQR**	***n***	**Median**	**IQR**	***n***	***P*****-value LC vs NC**
Any symptom	117	50–269	963	91	49–184	153	124	51–282	753	0.037
Coughing up blood	55	24–124	53	91	29–106	7	49.5	24–138	42	0.587
Cough or worsening cough >3 weeks	118	60–247	459	127	59–184	61	119.5	61–256	382	0.497
Breathlessness or worsening >3 weeks	129.5	66–305	256	90	57–286	40	154	73–319	199	0.161
Chest/shoulder pain >3 weeks	101.5	57–203	142	69	49–126	24	120.5	71–261	110	**0.044**
Hoarseness >3 weeks	101	52–253	89	102.5	59–266	10	113	51–255	75	0.759
Decreased appetite	98	46–197	87	66	52–154	18	101.5	43–200	60	0.316
Unexplained weight loss	177	85–379	46	126	59–369	11	216	94–382	30	0.377
Fatigue or tiredness ‘unusual for you'	118	56–246	231	77.5	58–177	40	126	53–267	179	0.262
Different ‘in yourself'	102.5	56–256	212	65.6	57–158	28	114.5	54–273	168	0.136
Other	93	49–179	167	63	38–107	22	97	47–199	141	0.065

Abbreviation: IQR=interquartile range.

Values are days unless otherwise stated.

a Analysing all cancer (LC plus OC) compared with no cancer (NC) produced similar findings, although breathlessness was also associated with a shorter time to diagnosis (LC 89 days (57–197) *vs* NC 154 days (73–319), *P*=0.049).

**Table 4:** Predictors of total diagnostic interval stratified by lung cancer and no cancer groups

	**Total cohort (*****n*****=693)**		**Primary lung cancer (LC) (*****n*****=153)**		**No cancer (NC) (*****n*****=753)**	
**Variable**	**Adjusted HR (95% CI)**	***P*****-value**	**Adjusted HR (95% CI)**	***P*****-value**	**Adjusted HR (95% CI)**	***P*-value**
Age (years)	1.00 (0.99–1.01)	0.79	1.00 (0.97–1.02)	0.75	1.00 (0.99–1.01)	0.81
Gender (reference: female)	0.91 (0.79–1.05)	0.21	1.06 (0.69–1.63)	0.79	0.90 (0.77–1.05)	0.19
**Educational status (reference: degree or higher)**						
A level/GCSE/O level	0.85 (0.71–1.01)	0.06	0.72 (0.44–1.19)	0.20	0.91 (0.75–1.11)	0.36
Other/none	0.88 (0.73–1.06)	0.17	0.66 (0.42–1.05)	0.08	0.93 (0.75–1.16)	0.54
**Smoking status (reference: smoker)**						
Ex-smoker	0.92 (0.73–1.16)	0.50	1.29 (0.79–2.10)	0.32	0.88 (0.67–1.17)	0.40
Never smoker	0.83 (0.65–1.06)	0.14	1.27 (0.53–3.00)	0.59	0.86 (0.64–1.15)	0.31
Missing	0.30 (0.04–2.21)	0.24	—		0.32 (0.04–2.38)	0.26
**Deprivation IMD (reference=1st quintile ‘least deprived')**						
2nd Quintile	1.19 (0.98–1.43)	0.08	0.98 (0.58–1.65)	0.94	1.18 (0.95–1.47)	0.14
3rd Quintile	0.98 (0.80–1.20)	0.84	1.34 (0.77–2.34)	0.30	0.91 (0.72–1.15)	0.44
4th Quintile	1.03 (0.82–1.30)	0.80	1.43 (0.78–2.63)	0.25	0.97 (0.74–1.26)	0.80
5th Quintile	0.96 (0.77–1.20)	0.71	0.87 (0.47–1.64)	0.68	0.95 (0.74–1.23)	0.71
**Comorbidity**						
Respiratory	0.85 (0.72–0.99)	0.04	0.78 (0.49–1.25)	0.31	0.85 (0.71–1.02)	0.07
Perceived family history cancer risk	0.95 (0.82–1.11)	0.55	0.76 (0.50–1.18)	0.22	0.94 (0.79–1.12)	0.50
**First symptom/s**						
Coughing up blood	2.17 (1.63–2.89)	0.00	Not included^a^		2.41 (1.74–3.35)	0.00
Cough or worsening cough >3 weeks	1.35 (1.17–1.56)	0.00	1.16 (0.78–1.74)	0.46	1.42 (1.20–1.67)	0.00
Breathlessness or worsening >3 weeks	0.96 (0.83–1.12)	0.62	0.70 (0.45–1.08)	0.10	1.00 (0.84–1.18)	0.97
Chest/shoulder pain >3 weeks	1.16 (0.96–1.41)	0.12	1.79 (1.08–2.99)	0.03	1.05 (0.85–1.31)	0.63
Hoarseness >3 weeks	1.20 (0.95–1.52)	0.12	0.98 (0.48–2.01)	0.97	1.24 (0.96–1.61)	0.10
Decreased appetite	1.54 (1.22–1.95)	0.00	1.41 (0.78–2.53)	0.25	1.54 (1.16–2.05)	0.00
Unexplained weight loss	0.79 (0.58–1.08)	0.14	0.86 (0.43–1.71)	0.66	0.69 (0.47–1.02)	0.06
Fatigue or tiredness ‘unusual for you'	1.17 (0.99–1.39)	0.06	1.16 (0.75–1.79)	0.49	1.18 (0.96–1.43)	0.11
Different ‘in yourself'	1.18 (0.99–1.41)	0.06	1.52 (0.93–2.46)	0.09	1.19 (0.97–1.45)	0.10

Abbreviations: CI=confidence interval; HR=hazard ratio; IMD=Index of Multiple Deprivation.

a Less than 10 cases.

**Table 5:** Predictors of total diagnostic interval stratified by lung cancer stage

	**Early stage (*****n*****=48)**		**Late stage (*****n*****=103)**	
**Variable**	**Adjusted HR (95% CI)**	***P*****-value**	**Adjusted HR (95% CI)**	***P*****-value**
Age	0.98 (0.93–1.03)	0.39	1.01 (0.98–1.04)	0.53
Gender (reference: female)	2.87 (1.01–8.16)	0.05	0.90 (0.51–1.56)	0.70
**Educational status (reference: degree or higher)**				
A level/GCSE/O level	0.40 (0.13–1.23)	0.11	0.76 (0.41–1.41)	0.39
Other/none	0.44 (0.16–1.17)	0.10	0.63 (0.36–1.12)	0.12
**Smoking status (reference: smoker)**				
Ex-smoker	1.52 (0.42–5.48)	0.52	1.04 (0.58–1.88)	0.89
Never smoker	1.32 (0.33–5.24)	0.70	1.02 (0.25–4.13)	0.98
**Deprivation IMD (reference=1st quintile ‘least deprived')**				
2nd Quintile	0.87 (0.30–2.50)	0.79	1.21 (0.62–2.40)	0.57
3rd Quintile	2.33 (0.61–8.92)	0.22	1.15 (0.59–2.25)	0.68
4th Quintile	1.36 (0.33–5.63)	0.67	1.29 (0.62–2.66)	0.50
5th Quintile	2.70 (0.55–13.28)	0.22	0.75 (0.36–1.56)	0.44
**Comorbidity**				
Respiratory	0.97 (0.40–2.396)	0.95	0.63 (0.34–1.18)	0.15
Perceived family history cancer risk	0.18 (0.06–0.51)	0.00	1.17 (0.67–2.03)	0.58
**First symptom/s**				
Coughing up blood	Not included^a^	—	Not included^a^	—
Cough or worsening cough >3 weeks	0.58 (0.21–1.57)	0.28	1.07 (0.66–1.72)	0.79
Breathlessness or worsening >3 weeks	0.24 (0.08–0.74)	**0.01**	0.96 (0.55–1.70)	0.90
Chest/shoulder pain >3 weeks	Not included^a^	—	1.66 (0.90–3.08)	0.11
Hoarseness >3 weeks	Not included^a^	—	Not included^a^	—
Decreased appetite	Not included^a^	—	1.29 (0.68–2.41)	0.43
Unexplained weight loss	Not included^a^	—	Not included^a^	—
Fatigue or tiredness ‘unusual for you'	0.40 (0.13–1.20)	0.10	1.26 (0.72–2.20)	0.42
Different ‘in yourself'	2.53 (0.81–7.90)	0.11	1.59 (0.84–3.01)	0.15

Abbreviations: CI=confidence interval; HR=hazard ratio; IMD=Index of Multiple Deprivation.

a Less than 10 cases.

**Table 6:** Multivariable model of predictors of primary lung cancer diagnosis

**Variable**	**Adjusted OR**	**95% CI**	***P*****-value**
Age	1.05	1.03–1.07	0.00
**Smoking status (ref=smoker)**			
Ex-smoker	0.41	0.25–0.67	0.00
Never smoker	0.06	0.03–0.14	0.00
Coughing up blood	1.85	1.14–2.99	0.01
Respiratory comorbidity	0.64	0.42–0.99	0.045
Arthritis comorbidity	0.53	0.34–0.82	0.00

Abbreviations: CI=confidence interval; OR=odds ratio.

## Supplementary Material

Supplementary Information

Supplementary Table A1

Supplementary Table A2
