# Supplementary material for: Symptoms and other factors associated with time to diagnosis and stage of lung cancer: a prospective cohort study
Source: Br J Cancer. 2015 Mar 3;112(Suppl 1):S6–S13. doi: 10.1038/bjc.2015.30 (PMC4385970; doi:10.1038/bjc.2015.30)
Supplement: Supplementary Information [file bjc201530x1.docx]

Table A1: Time to diagnosis (days) for first symptom/s among primary lung cancer group, stratified by stage (i) all; (ii) excluding cases where symptom duration was 28 days or less (Waiting time paradox sensitivity analysis)

Table A2: Univariable analyses of predictors of primary lung cancer diagnosis
