# Supplementary material for: Symptoms and other factors associated with time to diagnosis and stage of lung cancer: a prospective cohort study
Source: Br J Cancer. 2015 Mar 3;112(Suppl 1):S6–S13. doi: 10.1038/bjc.2015.30 (PMC4385970; doi:10.1038/bjc.2015.30)
Supplement: Supplementary Table A1 [file bjc201530x2.docx]

**Table A1: Time to diagnosis (days) for first symptom/s among primary lung cancer group, stratified by stage**

**(i) all; (ii) excluding cases where symptom duration was 28 days or less (Waiting time paradox sensitivity analysis)**

|  | **Early Stage**  **(n = 48)** | | | **Late Stage**  **(n = 103)** | | **p value** |
| --- | --- | --- | --- | --- | --- | --- |
|  | **Median (IQR)** | **n** | **Median (IQR)** | | **n** |  |
| **(i) ALL** | | | | | | |
| **Any Symptom** | **141 (52.5 - 253)** | **48** | **87 (51-175)** | | **103** | **0.33** |
| Coughing up blood | 154 (154 – 154) | 1 | 81 (29 – 98) | | 6 | 0.13 |
| Cough or worsening cough >3 weeks | 168 (65 – 205) | 15 | 108.5 (60 – 176) | | 44 | 0.36 |
| Breathlessness or worsening >3 weeks | 238 (66 – 407) | 10 | 71 (57 – 173) | | 29 | 0.23 |
| Chest/shoulder pain > 3 weeks | 115.5 (65 – 195) | 6 | 63 (46 – 106) | | 18 | 0.23 |
| Hoarseness > 3 weeks | 266 (191 – 321) | 3 | 69 (58– 107) | | 7 | 0.09 |
| Decreased appetite | 83 (23 – 168) | 3 | 64 (52 – 154) | | 15 | 0.95 |
| Unexplained weight loss | 197.5 (124- 271) | 2 | 126 (59 – 369) | | 9 | 0.81 |
| Fatigue or tiredness ‘unusual for you’ | 140 (46 - 275) | 11 | 71 (59 – 154) | | 29 | 0.45 |
| Different ‘in yourself’ | 74.5 (56 – 271) | 10 | 63.5 (57 – 126) | | 18 | 0.46 |
| **(ii) EXCLUDING CASES WHERE SYMPTOM DURATION <=28 DAYS** | | | | | | |
| **Any Symptom** | **168 (78 - 275)** | **39** | **106 (58-179)** | | **94** | **0.02** |
| Coughing up blood | 154 (154 – 154) | 1 | 91 (71 – 98) | | 5 | 0.14 |
| Cough or worsening cough >3 weeks | 168 (66 – 205) | 14 | 110 (60 – 180) | | 43 | 0.22 |
| Breathlessness or worsening >3 weeks | 307 (89 – 407) | 9 | 81 (57.5 – 185) | | 28 | 0.10 |
| Chest/shoulder pain > 3 weeks | 115.5 (65 – 195) | 6 | 63 (46 – 106) | | 18 | 0.23 |
| Hoarseness > 3 weeks | 266 (191 – 321) | 3 | 69 (58– 107) | | 7 | 0.09 |
| Decreased appetite | 125.5 (83 – 168) | 2 | 64 (52 – 154) | | 15 | 0.30 |
| Unexplained weight loss | 197.5 (124- 271) | 2 | 126 (59 – 369) | | 9 | 0.81 |
| Fatigue or tiredness ‘unusual for you’ | 205 (78 - 275) | 9 | 71 (59 – 154) | | 29 | 0.07 |
| Different ‘in yourself’ | 83 (65 - 271) | 9 | 63.5 (57 – 126) | | 18 | 0.21 |
