# Supplementary material for: Symptoms and other factors associated with time to diagnosis and stage of lung cancer: a prospective cohort study
Source: Br J Cancer. 2015 Mar 3;112(Suppl 1):S6–S13. doi: 10.1038/bjc.2015.30 (PMC4385970; doi:10.1038/bjc.2015.30)
Supplement: Supplementary Table A2 [file bjc201530x3.docx]

**Table A2: Univariable analyses of predictors of primary lung cancer diagnosis**

| **Variable** | | | **Adjusted OR** | **95%CI** | **P -value** |
| --- | --- | --- | --- | --- | --- |
| **Age** | | | **1.04** | **1.01 – 1.07** | **0.00** |
| **Gender** (reference: female) | | | 0.94 | 0.62 – 1.43 | 0.79 |
| **Educational status**  (reference: degree or higher) | | A Level/GCSE/O Level | 0.88 | 0.52 – 1.48 | 0.64 |
|  |  | Other/ None | 1.18 | 0.71 – 1.98 | 0.51 |
| **Smoking status**  (reference: smoker) | | Ex-smoker | **0.48** | **0.28 – 0.82** | **0.01** |
|  |  | Never smoker | **0.07** | **0.03 – 0.16** | **0.00** |
| **Deprivation** IMD  (reference: 1^st^ quintile ‘least deprived’) | | 2^nd^ quintile | 1.45 | 0.84 - 2.50 | 0.19 |
|  |  | 3^rd^ quintile | 1.30 | 0.71 - 2.37 | 0.40 |
|  |  | 4^th^ quintile | 1.50 | 0.80 – 2.80 | 0.21 |
|  |  | 5^th^ quintile | 0.94 | 0.47 - 1.89 | 0.87 |
| **Employment** status  (reference: employed) | | Unemployed | 0.29 | 0.03 – 2.59 | 0.27 |
|  |  | Retired | 0.91 | 0.47 – 1.70 | 0.74 |
|  |  | Sick/disabled | 0.94 | 0.18 – 4.88 | 0.94 |
|  |  | Other (incl missing) | 0.70 | 0.22 – 2.19 | 0.54 |
| Not living alone | | | 0.81 | 0.54 – 1.20 | 0.29 |
| Ethnicity | | | 3.43 | 0.38 – 30.90 | 0.27 |
| **Co-morbidity** | Respiratory | | **0.62** | **0.40 – 0.98** | **0.04** |
|  | Arthritis | | **0.52** | **0.33 – 0.83** | **0.01** |
| **First symptom/s** | Coughing up blood | | **1.84** | **1.10- 3.05** | **0.02** |
|  | Cough or worsening cough >3 weeks | | 0.85 | 0.56 – 1.29 | 0.44 |
|  | Breathlessness or worsening >3 weeks | | 0.95 | 0.62 - 1.45 | 0.82 |
|  | Chest/shoulder pain > 3 weeks | | 1.50 | 0.95 – 2.35 | 0.08 |
|  | Hoarseness > 3 weeks | | 0.74 | 0.41 - 1.32 | 0.31 |
|  | Decreased appetite | | 1.23 | 0.70 – 2.16 | 0.47 |
|  | Unexplained weight loss | | 1.53 | 0.81 – 2.90 | 0.19 |
|  | Fatigue or tiredness ‘unusual for you’ | | 1.19 | 0.74 – 1.91 | 0.47 |
|  | Different ‘in yourself’ | | 0.87 | 0.53 - 1.42 | 0.57 |
